# Modern Evaluation of Liquisolid Systems with Varying Amounts of Liquid Phase Prepared Using Two Different Methods

**DOI:** 10.1155/2015/608435

**Published:** 2015-05-17

**Authors:** Barbora Vraníková, Jan Gajdziok, David Vetchý

**Affiliations:** Department of Pharmaceutics, Faculty of Pharmacy, University of Veterinary and Pharmaceutical Sciences, Palackého 1/3, 612 42 Brno, Czech Republic

## Abstract

Liquisolid systems are an innovative dosage form used for enhancing dissolution rate and improving *in vivo* bioavailability of poorly soluble drugs. These formulations require specific evaluation methods for their quality assurance (e.g., evaluation of angle of slide, contact angle, or water absorption ratio). The presented study is focused on the preparation, modern *in vitro* testing, and evaluation of differences of liquisolid systems containing varying amounts of a drug in liquid state (polyethylene glycol 400 solution of rosuvastatin) in relation to an aluminometasilicate carrier (Neusilin US2). Liquisolid powders used for the formulation of final tablets were prepared using two different methods: simple blending and spraying of drug solution onto a carrier in fluid bed equipment. The obtained results imply that the amount of liquid phase in relation to carrier material had an effect on the hardness, friability, and disintegration of tablets, as well as their height. The use of spraying technique enhanced flow properties of the prepared mixtures, increased hardness values, decreased friability, and improved homogeneity of the final dosage form.

## 1. Introduction

Bioavailability of drugs after oral administration depends on several factors such as aqueous solubility, drug permeability, dissolution rate, first-pass and presystemic metabolism, and susceptibility to efflux mechanisms. Poor solubility and low permeability represent the most frequent causes of limited bioavailability for a number of drugs. Solubility is the most important parameter for orally administered drugs which enable them to achieve the required concentration in systemic circulation necessary for the desired pharmacological response. The improvement of drug solubility remains one of the most challenging aspects of the drug development process especially for solid dosage forms designated for systemic absorption of the drug after oral administration [1]. Therefore, one of the most important and promising areas of the modern pharmaceutical technology is focused on modern approaches to the formulation and evaluation of solid dosage forms with enhanced bioavailability of poorly soluble drugs. These drugs represent up to 40% of commonly used active substances and almost 70% of newly synthesized molecules. Scientific literature describes a number of different techniques for improving the solubility and bioavailability of mentioned drugs (such as reducing particle size via micronization [2], using surfactants [3], lyophilization [4], and the preparation of self-emulsifying drug delivery systems [5]). Of all these, the formulation of liquisolid systems (LSS) represents one of the most promising and innovative techniques for promoting dissolution rate and* in vivo* bioavailability of poorly soluble drugs.

Liquisolid systems essentially refer to formulations prepared by converting a liquid drug or a drug in liquid state (solutions, suspensions, or emulsions) into dry, nonadherent, free-flowing, and readily compressible powder mixtures by blending or spraying a liquid dispersion onto specific powder carriers and coating materials [6]. The prepared dry blends can be subsequently transformed into conventional solid dosage forms (filled into capsules and compressed into tablets) which represents one important advantage of these systems [7]. Various grades of cellulose, a granulated form of magnesium aluminometasilicates (Neusilin), and a specifically prepared form of anhydrous dibasic calcium phosphate (Fujicalin) may be used as the carriers while very fine powders, such as colloidal silica or a powdered form of magnesium aluminometasilicates, could be used as coating materials.

Due to the their advantages, a number of poorly soluble drugs (such as atorvastatin [8], carbamazepine [9], furosemide [10], and indomethacin [11]) have been formulated as liquisolid systems to ensure enhanced drug release and improved bioavailability of active ingredients. Several mechanisms of enhanced drug release from liquisolid systems have been described in scientific literature. Increased surface area of the available drug and the drug in dissolved state represents the most important one of these. The drug within the liquisolid system is usually already dissolved in a nonvolatile solvent (propylene glycol, polyethylene glycol, glycerol, etc.) which keeps the drug from having to dissolve in the gastrointestinal tract, which is the most limiting step during drug absorption. Moreover, the solubilized drug in its molecularly dispersed state is still fixed on the large surface of the carrier material available for GI fluids [7, 12, 13].

The improved wetting properties of the liquisolid tablets by the dissolution media represent another one of the proposed mechanisms of the enhanced dissolution rate in liquisolid systems. The nonvolatile solvents used for liquisolid system formulations facilitate the wetting of the final solid dosage form by decreasing interfacial tension between dissolution medium and tablet/powder surface [14]. Improved wettability of these systems is usually demonstrated by measuring contact angles and water rising times (wetting times) [12, 14]. The improved wettability was proved, for example, by V. B. Yadav and A. V. Yadav [15]. In their study, they claimed that liquisolid granules containing indomethacin showed a significantly shorter rising time of water in comparison to raw indomethacin and also granules prepared using the compression (dry granulation) technique. This finding can be explained by the fact that water poorly soluble drug is, in the hydrophilic dissolved form (polyethylene glycol 400 solution), absorbed in the powder particles of the carrier of the liquisolid formulation [15].

In addition to the first two mentioned mechanisms of drug release enhancement, it could be expected that the solubility of the drug might be increased through the use of a suspension when formulated as a liquisolid system. In fact, the relatively small amount of liquid vehicle in a liquisolid compact is not sufficient to increase the overall solubility of the drug in an aqueous dissolution medium. However, it could also be expected that, in the microenvironment of the solid/liquid interface between an individual liquisolid primary particle and the release medium, the amount of the liquid vehicle diffusing out of a single liquisolid particle together with the drug molecules is sufficient to increase the aqueous solubility of the active ingredient by acting as a cosolvent [7, 12, 16].

In addition to conventional evaluation methods, specific tests for assessing liquisolid systems' quality parameters can be used (e.g., angle of slide and water absorption ratio or contact angle). Angle of slide (*θ*) is a specific parameter used to evaluate the flow properties of powder excipients. During such tests, an angle of 33° is regarded as optimal flow behavior for an LSS powder mixture [17]. To evaluate the improved wettability of the final liquisolid formulation, contact angle or water absorption ratio tests are performed. The contact angle is calculated by measuring the height and diameter of drop of dissolution medium placed on the tablet surface [6]. Water absorption ratio is related to the wetting time (time necessary for complete wetting of the tablet) and refers to the amount of water (in %) absorbed by the tablet during wetting [18].

Drugs from the group of hypolipidemic agents represent one of the best selling drugs. Mixed dyslipidemia, a common lipid abnormality characterized by altered levels of lipids and lipoproteins in blood plasma, is associated with increased risk of coronary heart disease which has been identified as the leading cause of death in developed countries [19, 20]. Statins, selective inhibitors of 3-hydroxy-3-methylglutaryl coenzyme A (HMG-CoA) reductase, are first-line drugs in the treatment of hypercholesterolemia due to their lowering effect on LDL-cholesterol [21]. Compared with other statins (lovastatin, simvastatin, fluvastatin, cerivastatin, and atorvastatin), rosuvastatin has the greatest amount of bonding interaction with HMG-CoA reductase and exhibits the minimal metabolization via cytochrome P450 3A4, the isoenzyme implicated in a wide variety of drug-drug interactions [22, 23]. However, rosuvastatin is poorly soluble in water which leads to low estimated absorption (~50%) and inadequate absolute bioavailability (~20%) [24].

The presented study is focused on the preparation,* in vitro* testing, and evaluation of differences of liquisolid systems containing varying amounts of liquid state drug (polyethylene glycol 400 solution of rosuvastatin) in relation to aluminometasilicate carrier Neusilin US2. Liquisolid powders used for the formulation of final tablets were prepared using two different methods: simple blending and spraying of drug dispersion onto carrier material in fluid bed equipment. The effect of the amount of the drug in liquid state and the method used for preparing liquisolid systems on their quality parameters were studied and evaluated with the aim of finding a formulation most suitable for* in vivo* testing.

## 2. Materials and Methods

### 2.1. Materials

Modern hypolipidemic agent, rosuvastatin calcium (Jai Radhe Sales, India), was used as the model drug. Polyethylene glycol 400 (Dr. Kulich Pharma, Czech Republic), Neusilin US2 (Fuji Chemical Industry Co., Ltd., Japan), Aerosil 200 (Eurošarm spol. s.r.o., Czech Republic), Lactose DCL 11 (DMV International GmbH, the Netherlands), and magnesium stearate (Zentiva a.s., Czech Republic) were used as the nonvolatile solvent, carrier, coating material, filler, and lubricant, respectively. Superdisintegrant Kollidon CL-F was received as a gift from BASF SE (Germany).

### 2.2. Methods 

#### 2.2.1. Preparation of Liquisolid Powders and Formulation of Tablets

Liquisolid powder blends were prepared by simple blending using mortar and pestle and/or by spraying in fluid bed equipment (Glatt AG, Switzerland). Rosuvastatin was dissolved in polyethylene glycol 400 (PEG 400) to obtain a 7.5% (w/w) solution (experimentally measured saturated concentration at 20°C). The resulting solution was applied to a precisely calculated amount of Neusilin US2 (carrier) and coated with Aerosil 200 (coating material) to obtain a dry powder with sufficient flow properties for further processing.

Powder materials used for the formulation of LSS can retain only a limited amount of liquid while maintaining acceptable flow and compression properties. Therefore, Spireas and Bolton [25, 26] established a mathematical approach for calculating the required amounts of carriers and coating materials. According to the previous study [27], the maximum liquid load factor (*L*_*f*_) (the ratio of the weight of the drug in liquid state to the carrier material weight) was determined to be 1.2 and the excipient ratio (*R*) (the weight ratio of the carrier material to the coating material) was determined to be 50. The appropriate quantities of carrier (*Q*) and coating material (*q*) required to transform a given amount of drug in liquid state (*m*) into an acceptably flowing and compressible liquisolid system were calculated from the following equation:

(1) $$\begin{array}{c} L_{f}=\frac{m}{Q},\\ R=\frac{Q}{q}. \end{array}$$

In the case of simple blending, the liquid form of the drug was mixed with a calculated amount of carrier and coating material. The blend was passed through a sieve (mesh size 1 mm) and subsequently mixed in a three-axial homogenizer (T2C, TURBULA System Schatr, Switzerland) for 10 minutes. Lactose and Kollidon CL-F were added; the mixture was sieved (mesh size 1 mm) and homogenized in the homogenizer for another 10 minutes. At the end, magnesium stearate (a lubricant) was added and the whole blend was sieved (mesh size 1 mm) and mixed for 2 more minutes.

For the fluid-bed spraying method, the drug solution was sprayed onto Neusilin US2, and the mixture was then passed through a sieve (mesh size 1 mm) and mixed in a homogenizer for 10 minutes. After this, Aerosil 200 was added and the whole mixture was sieved (mesh size 1 mm) and mixed for 5 minutes. Lactose and Kollidon CL-F were then added, and powder blend was sieved (mesh size 1 mm) and mixed for another 10 minutes. For the final part of the preparation procedure, magnesium stearate was added, and the mixture was sieved (mesh size 1 mm) and mixed for 2 more minutes.

Oblong tablets (18 × 8 mm) with constant weight of 650 mg were directly compressed from the prepared dry blends using an excentric tablet press (EK 0, KORSCH, Germany). The prepared tablets were kept in polyethylene bag for 48 hours before testing.

Samples were marked according to the representation (percentage w/w) of the drug in liquid state in relation to the carrier (Table 1). For example, sample 60%w contained 60% liquid phase in relation to the weight of Neusilin US2.

#### 2.2.2. Powder Flow of the Liquisolid Tableting Mixtures

The flow properties of prepared liquisolid tableting mixtures were established by determining the flowability (flow through the orifice), angle of repose, compressibility index, and Hausner ratio. A defined stainless steel funnel with an orifice of 2.5 cm in diameter and a fixed glass funnel were used to measure the flowability and angle of repose as implies Ph. Eur. 8.0. Bulk and tapped densities were determined also according to Ph. Eur. 8.0 for the calculation of Hausner ratio (HR) and compressibility index (CI) [28].

#### 2.2.3. Angle of Slide

**Figure 1:**
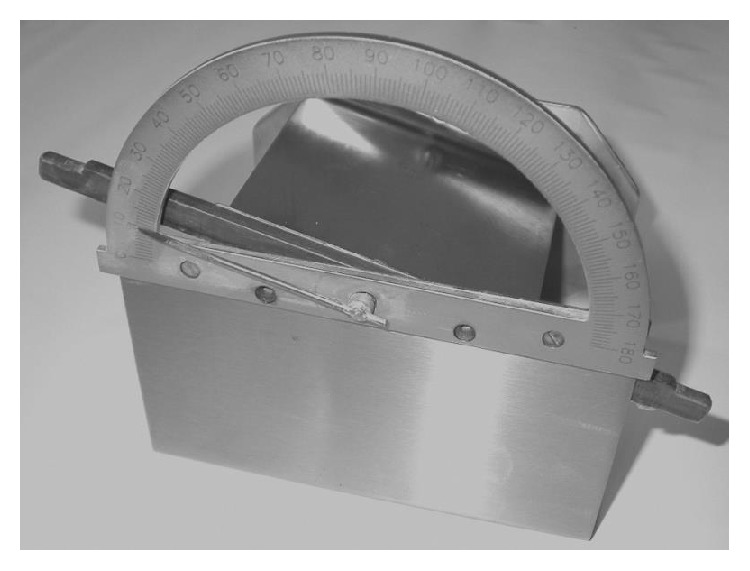
Equipment for evaluation of angle of slide.

Angle of slide is a specific parameter for evaluating the flow behaviour of liquisolid mixtures [29]. Angle of slide was used to evaluate the flow properties of liquisolid tableting mixtures. The tested powder sample (10 g) was placed on one end of a metal plate with a polished surface (Figure 1). This end was gradually raised until the plate with the horizontal surface formed an angle at which the sample was about to slide. The measurement was repeated 3 times; average and standard deviations were calculated. Angle of slide corresponding to 33° is regarded as optimal flow behaviour [16].

#### 2.2.4. Pycnometric Density

The density of tableting mixtures was evaluated using the gas displacement technique with a helium pycnometer (PYCNOMATIC ATC, POROTEC, Germany). An accurately weighed and completely dry test cell was filled with the powder sample and weighed again. The test cell containing the sample was sealed in the pycnometer and analysis commenced. Each sample was measured three times and average and standard deviations were calculated.

#### 2.2.5. Tablet Hardness

Hardness of liquisolid tablets (the force in Newton required to crush the tested tablet) was evaluated using a hardness tester (C 50 Tablet Hardness & Compression tester, ENGINEERING SYSTEMS (Nottm) Ltd., UK). Ten randomly selected oblong tablets of each formulation were tested in both directions (transversely and lengthwise); mean values and standard deviations were calculated.

#### 2.2.6. Friability

Approximately 6.5 g of dedusted tablets was weighed precisely using an analytical balance (KERN 870-13, Gottl. KERN & Sohn, Germany) and placed into the plastic drum of an abrasion tester (TAR 10, ERWEKA GmbH, Germany) and rotated for 4 minutes at 25 rpm, corresponding to Ph. Eur. 8.0. Afterwards, the dust was removed and tablets were reweighed. The loss of mass in each tablets' sample was determined. Percentages were calculated using the following equation [28]:

(2) $$\begin{array}{c} \%\;\text{Friability}=\frac{\text{loss}\;\text{of}\;\text{mass}}{\text{initial}\;\text{mass}}*100. \end{array}$$

#### 2.2.7. Disintegration

The disintegration test was performed at 37.0 ± 2.0°C in distilled water on six tablets from each formulation using a disintegration test apparatus (ZT4, ERWEKA GmbH, Germany). The tablets were considered completely disintegrated when no residue remained in the basket. The presented values are the means and SDs of six determinations.

#### 2.2.8. Uniformity of Mass

From each formulation, 10 randomly selected tablets were weighed individually on an analytical balance (KERN 870-13, Gottl. KERN & Sohn, Germany). The average weight of all tablets and percentage deviation from the mean value for each tablet were determined.

#### 2.2.9. Drug Content

For this process, 10 randomly selected representative tablets from each batch were evaluated for their drug content. 500 mL of distilled water was added to each tablet to dissolve the drug. The dispersion was kept at laboratory temperature (20°C) for at least 3 hours. Samples were filtered and then analysed spectrophotometrically (LAMBDA 25, PERKIN ELMER INSTRUMENTS, USA) at 242 nm. The percentages of individual drug content were calculated and compared to the theoretical drug content (10 mg).

#### 2.2.10. Tablet Height

The heights of 3 tablets were measured using a digital slide caliper (DS 150, QUANTUM MASCHINEN, Germany). Measurement was carried out 5 times for each batch of tablets. The average weight of one tablet and the standard deviation of measurement were calculated.

#### 2.2.11. Determination of Wetting Time and Water Absorption Ratio

Wetting time and water absorption ratio of liquisolid tablets were determined in a Petri dish using a sponge (5 × 5 cm), impregnated by ten grams of water containing a water-soluble green colour (brilliant green) for identification of complete tablet surface wetting. Tested liquisolid tablet was carefully placed on the surface of the impregnated sponge in the Petri dish at laboratory temperature (20°C). The time required to reach the upper surface of the tablet by the colour solution was noted as the wetting time (time necessary for complete wetting of the tablet). The weight of the tablet in the dry state (before being placed on the sponge) was noted as *m*_0_. The wetted tablet was removed and reweighed (*m*_1_). The water absorption ratio (WA) was calculated using the equation:

(3) $$\begin{array}{c} WA=100*\frac{\left(m_{1}-\;m_{0}\right)}{m_{0}}. \end{array}$$

The measurement was carried out 5 times for each batch of tablets; results are presented as mean values and standard deviations.

#### 2.2.12. *In Vitro* Dissolution Studies

*In vitro* release of rosuvastatin was determined using a standard paddle dissolution apparatus (Sotax AT 7 Smart, Sotax, Switzerland) with a paddle speed of 50 rpm in 500 mL of artificial gastric fluid (pH 1.2) at 37.0 ± 0.5°C. Throughout the experiment, the withdrawn samples were analysed spectrophotometrically online at 242 nm at time intervals 5, 10, 15, 20, 25, and 30 min. Six randomly selected tablets of each formulation were tested; results are presented as mean values and standard deviations.

## 3. Results and Discussion

### 3.1. Powder Flow of the Liquisolid Tableting Mixtures

Currently, a number of various methods are used for characterizing the flow properties of pharmaceutical powder materials. Therefore, several parameters, such as flowability, angle of repose, angle of slide, compressibility index, and the Hausner ratio, were used to determine the flow properties of prepared liquisolid powder mixtures. Angle of slide is a specific parameter used to evaluate flow properties of prepared liquisolid powdered blends. Spireas et al. [30] claimed that angle of slide is the preferred method to determine the flowability of powders with particles smaller than 150 *μ*m.

The evaluation of flowability (Table 2) of powdered mixtures prepared by simple blending implied that increasing the amount of liquid phase in relation to aluminometasilicate carrier Neusilin US2 improved the flowability of the prepared powder blend. The flowability improvement is a result of the increased weight of the Neusilin US2 granules with sorbed rosuvastatin solution, hence decreasing the pycnometric density of the liquisolid tableting mixtures (Table 3). The decreased pycnometric density with the increasing amount of liquid phase in relation to the carrier material could be explained, as Neusilin pores became filled with liquid. Gumaste et al. in their study [31] proved that the majority of the liquid is adsorbed into mesopores and deep into the channels of the Neusilin US2's macropores. Hentzschel et al. [32] showed that the addition of liquid phase (tocopherol acetate) to Neusilin US2 decreased flowability. In general, the flow properties of the liquisolid blends were enhanced in comparison to Neusilin US2 alone [27, 32]. Moreover, samples with more liquid contained higher amounts of spray-dried lactose (DCL 11) as filler, with excellent flow properties [33], supporting their improved flowability. The flowability values of the powder blends prepared by spraying in fluid bed equipment did not indicate any dependence of the amount of the drug in the liquid state added to carrier. The highest flowability value (57.90 ± 0.90 s/100 g) was exhibited by sample 80%w and the lowest value (5.19 ± 0.01 s/100 g) by sample 90%w. Excluding samples 100%w, 110%w, and 120%w, flowability of the samples prepared by spraying was lower in comparison to mixtures prepared by simple blending. This improvement of the flow properties could be explained by the improved homogeneity of blends obtained by spraying in fluid bed equipment [34].

Determination of angle of repose (Table 2) did not reveal any dependence on the amount of the used drug in liquid state or the used method. This finding supports the argument that measurement of angle of slide is more suitable for determining flow properties of liquisolid systems. The measured values of angle of repose for samples prepared by simple blending ranged between 26.43 ± 0.48° and 31.51 ± 0.87° and corresponded with excellent to good flow characteristics [28]. Blends obtained by spraying in fluid bed equipment showed values of angle of repose in range between 21.19 ± 0.53° and 30.70 ± 0.88° which responded to excellent flow properties in compliance with European Pharmacopoeia [28].

Results obtained from measuring angle of slide implied that sample 110%w prepared by simple blending and 120%w prepared by spraying exhibited the recommended angle of slide (about 33°) [16] (Table 2). Values lower than 33° were observed in only three samples prepared by spraying (40%w, 60%w, and 110%w). Other blends provided angle of slide values higher than the recommended angle of 33°, which indicated inferior flow properties of the blends. In general, mixtures prepared by spraying showed lower values of angle of slide than blends achieved by simple blending. This observation could be explained by the improved homogeneity of blends created by spraying in fluid bed equipment [34].

Evaluation of compressibility index and Hausner ratio (Table 2) implied that blends prepared by simple mixing exhibited fair flow characteristics in compliance with European Pharmacopoeia [28]. Tableting mixtures prepared by spraying provided lower CI and HR values in comparison to blends obtained by simple blending. Their values corresponded to good and fair flow properties. The results confirmed previous studies dealing with liquisolid blends where HR values ranging between passable and excellent flow properties were observed after adding a liquid phase. The sorption of 0.9% solution of griseofulvin in PEG 300 [35] and olmesartan medoxomil in Acrysol EL 135 [36] onto carrier Neusilin US2 are two examples of this.

### 3.2. Tablet Hardness

**Figure 2:**
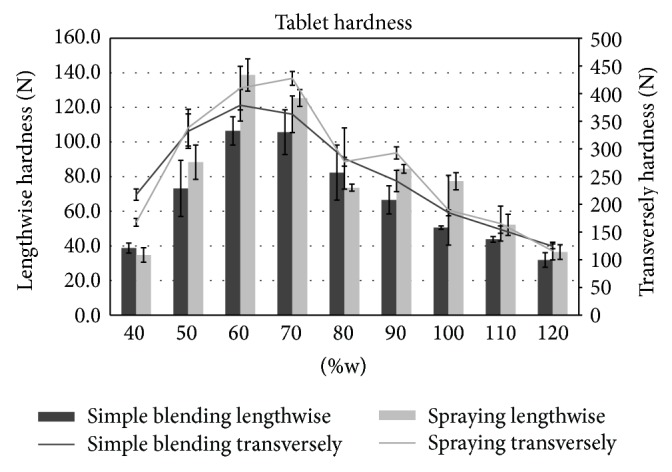
Tablet hardness.

Tablet hardness is one of the basic tests of final dosage form quality and mechanical durability. This parameter depends on a number of factors such as tablet shape, size, composition, and used compression force and equipment [37]. Therefore, prepared liquisolid tablets were evaluated in two directions (transversely and lengthwise) due to their oblong shape. All tablets were compressed to the experimentally adjusted maximum hardness. In general, it was observed that the hardness of the tablets placed between the hardness testers' jaws lengthwise was lower than those measured transversely (Figure 2). The obtained results implied that hardness increased initially with the increasing amount of liquid phase up to 60% in relation to carrier Neusilin US2 (Figure 2). However, from the 70% representation of drug in liquid phase, values decreased again in both measured directions (Figure 2). A similar tendency was observed by Hentzschel et al. [32] in their study, where Neusilin US2 was able to absorb up to 50% of tocopherol acetate while maintaining acceptable mechanical properties of the prepared tablets. The decreasing values of hardness could be explained by the squeezing of the liquid from the tablets structure during compression (liquid-squeezing out phenomena) and by the negative effect of liquid on the bonds between solid carrier particles necessary for adequate tablet quality [38]. Figure 2 illustrates that tablets compressed from the blends prepared by simple blending had a generally lower hardness in comparison to tablets from mixtures prepared by spraying, which could be related to better flowability and compressibility of these blends as shown in the results of the compressibility index (Table 2).

### 3.3. Friability

All the tested tablet samples showed friability values (Table 4) lower than the Ph. Eur. 8.2 limit, which for uncoated tablets is 1% [28]. The only exception to this was observed in sample 120%w prepared by simple blending (1.45%). This finding correlates with this sample's lower hardness values (31.9 ± 4.4 N and 124.35 ± 4.34 N). In general, it can be stated that the rest of the tablets, which fulfilled the requirements for friability, are expected to withstand the stress and attrition of common handling, packaging, and transporting processes.

### 3.4. Disintegration

Rapid tablet disintegration is necessary to ensure tablets' quick collapse into smaller fragments to obtain the largest possible surface area accessible for dissolution media [39]. The determined disintegration times of liquisolid tablets are shown in Table 4. The disintegration test revealed that all liquisolid system formulations disintegrated within a maximum of 159 ± 3 s, which complies with the specifications given for the uncoated tablets in the Ph. Eur. [28]. All the prepared liquisolid formulations could also be marked as fast disintegrating tablets which disintegrate rapidly (within 3 min) and could be beneficial for patients with difficulty in swallowing [28, 40]. The observed fast tablet disintegration could be explained by the presence of superdisintegrant Kollidon CL-F in combination with hydrophilic solvent PEG 400 which improves wetting properties of the liquisolid tablet and shortens its disintegration time [14]. Preparing fast disintegrating tablets could improve the bioavailability of rosuvastatin thanks to the fast and easy transition of the dissolved drug in a hydrophilic liquid (PEG 400) to GI fluids, from which the active ingredient could be absorbed into systemic circulation.

### 3.5. Uniformity of Mass

All the prepared liquisolid tablets met the requirements for the uniformity of mass test. None of the tablets deviated from the average value by more than 5% (Table 4), which is the limit given by European Pharmacopoeia [28]. The results proved that both methods of preparations led to the formulation of blends with good flow properties and processability and to tablets with homogeneous weight, as indicated by low values of standard deviations.

### 3.6. Drug Content

Fahmy and Kassem [13] claimed that the process of adsorption of liquid phase onto carriers provides uniform drug distribution in the final dosage form, thereby ensuring good content uniformity. However, evaluation of the drug content (Table 4) implied that samples 90%w (66.10 ± 3.83%), 100%w (116.76 ± 5.03%), 110%w (118.19 ± 13.15%), and 120%w (85.68 ± 23.70%) obtained by simple blending did not meet content uniformity criteria in compliance with Eur. Ph. [28]. This problem could be a result of inhomogeneous distribution of the drug solution in the tableting blends, as high values of standard deviation implied. This issue might be resolved by spraying the drug in liquid phase onto the carrier in fluid bed equipment or mixing the blends in high-shear mixers. The results of drug content evaluations of tablets prepared by spraying have proved this assumption, as shown in Table 4. Moreover, standard deviations of the tablets prepared by spraying (maximum value 5%) were lower in comparison to tablets obtained by simple blending (maximum value 23.70%).

### 3.7. Tablet Height

The amount of the drug in the liquid state presented in the formulation showed an impact also on tablet height. Initially, the height of the prepared liquisolid tablets decreased with increasing amount of liquid in relation to the carrier Neusilin US2 (from sample 40%w with 8.20 ± 0.07 mm to sample 70%w with 4.90 ± 0.04 mm). Subsequently, the height of tablets containing 70% and more liquid phase was very similar due to the saturation of carrier pores by liquid and the presence of higher amounts of lactose with limited compaction properties (Table 4). In addition, it was observed that the tablets' height was influenced by the formulation method. Tablets prepared from mixtures obtained by spraying had generally lower height values (6.22 ± 0.13 to 4.30 ± 0.00 mm) in comparison to tablets prepared by simple blending (8.20 ± 0.07 to 4.73 ± 0.08 mm). These results are related to the better compressibility of the liquisolid blends, which contain higher amounts of liquid in relation to carrier material and better flow properties of mixtures prepared by spraying in fluid bed equipment.

### 3.8. Wetting Time and Water Absorption Ratio

One mechanism that might explain the enhanced dissolution rate from the liquisolid systems is the improved wettability of the final liquisolid dosage form by the dissolution media (natural or artificial GI fluids). This is caused by hydrophilization of the solid particles' surface through the incorporation of hydrophilic liquid media (drug solution, emulsion, and suspension) [14]. As a result, water absorption ratio and time required for complete wetting of the tablet by aqueous media (wetting time) were evaluated (Table 5). The water absorption ratio of tablets prepared by simple blending ranged between 75.26 ± 11.03% (70%w) and 173.17 ± 12.60% (40%w). The absorption ratio initially decreased as the amount of drug in liquid state increased up to 90% in relation to Neusilin US2. After 90%, the ratio began to increase. An explanation for this could be the saturation of pores of the carrier particles in formulations with lower representation of the drug in liquid state (40%w–70%w). The subsequent slight increase of water uptake could be caused by the improved hydrophilicity of Neusilin particles surface which contains a higher amount of drug in hydrophilic liquid state, as well as a higher amount of lactose as hydrophilic filler in the formulation. Water absorption ratio of tablets obtained by spraying was generally lower compared to tablets prepared by simple blending.

The lower absorption ratios are related to the faster wetting time of these samples (1.67 ± 1.05–9.93 ± 2.78 min). In terms of tablets prepared by spraying, this parameter did not imply any evident dependence on the amount of drug in liquid phase in relation to the carrier material and testing of faster wetting time could be affected by measurement errors resulting in higher values of standard deviation (Table 5). The wetting time of tablets compressed from mixtures prepared by simple blending increased as the amount of Neusilin US2 in the formulation decreased. This prolongation of the wetting time could be caused by saturation of Neusilin particles pores by the drug solution, thus reducing its ability to absorb another liquid. The slowest wetting time was exhibited by sample 120%w (55.87 ± 4.77) prepared by simple blending and the fastest sample was 110%w (1.67 ± 1.05 min) prepared by spraying. The obtained values of wetting time were higher in comparison to wetting times measured by Kapure et al. [41]. In their study, rosuvastatin tablets containing microcrystalline cellulose as a carrier were completely wetted after 20 s. Wetting time is closely related to the inner structure of the tablets and to the hydrophilicity of the excipients used. Therefore, liquisolid systems containing microcrystalline cellulose with a fast water wicking rate [42] are wetted faster than those containing insoluble and less hydrophilic Neusilin US2 [35].

### 3.9. *In Vitro* Dissolution Studies

**Figure 3:**
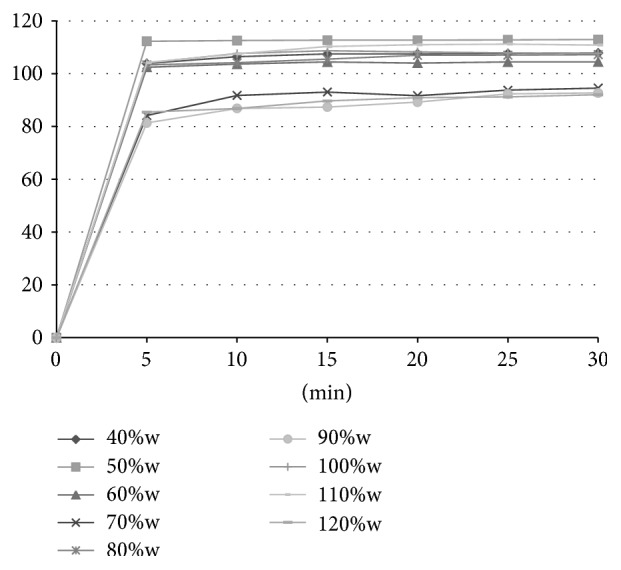
Dissolution profiles of tablets compressed from powders prepared by simple blending.

**Figure 4:**
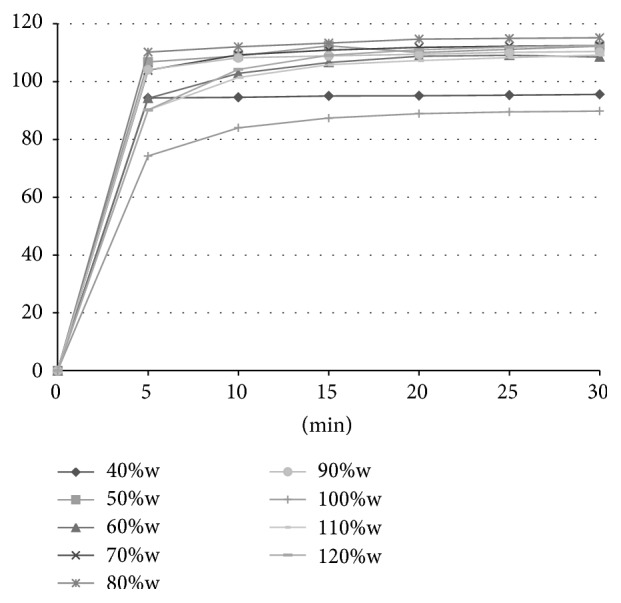
Dissolution profiles of tablets compressed from powders prepared by spraying in fluid bed equipment.

The dissolution profiles of rosuvastatin liquisolid tablets are presented in Figures 3 and 4, respectively. The percentage of rosuvastatin released from tablets prepared by simple blending during the first 5 minutes ranged between 81.34 ± 24.27% (90%w) and 112.27 ± 3.42% (50%w). Higher values of standard deviations of these measurements (Table 6) are related to poorer homogeneity of the tablets, as was previously mentioned. The similar dissolution profiles were obtained also for tablets prepared by spraying. The amount of rosuvastatin released from these compacts during the first 5 minutes ranged between 74.27 ± 2.30% (100%w) and 110.25 ± 1.39% (80%w). The release of rosuvastatin from all liquisolid tablets was faster in comparison to the results of the directly compressed tablets introduced by Kapure et al. [42]. In this study, the amount of drug released after 15 minutes from conventional rosuvastatin tablets was about 15% (as 450 mL of a pH 1.2 solution was used as a dissolution medium). This value is considerably lower in comparison to liquisolid tablets, which showed an 80% release of drug during the first five minutes of testing. The same enhanced release profile has already been proved in several studies dealing with liquisolid systems, such as those of liquisolid compacts containing clonazepam [43], candesartan cilexetil [44], and griseofulvin [35]. The enhanced release could be caused by the presence of a nonvolatile liquid vehicle which improved the wettability of the compacts and hence their disintegration time and by the presence of the drug in its dissolved form without the need to dissolve it in the dissolution medium [14].

## 4. Conclusion

Preparation of liquisolid systems is one of the most promising and innovative techniques for enhancing* in vitro* dissolution rate and improving* in vivo* bioavailability of poorly soluble drugs. A liquisolid system can be prepared by incorporating a drug in liquid state (liquid drug; drug solution, suspension, or emulsion) onto a specific carrier and coating material while forming a dry, free-flowing, and readily compressible powdered blend. Liquisolid systems are unique medical forms which require specific evaluation for their quality assurance. The presented work was aimed at the modern evaluation of liquisolid systems and the evaluation of differences among liquisolid tablets containing varying amounts of rosuvastatin solution in relation to a magnesium aluminometasilicate carrier (Neusilin US2). Liquisolid powder blends were prepared using two different methods: simple blending and spraying in fluid bed equipment. From the obtained results, it could be stated that all liquisolid tablets had very fast disintegration times connected to enhanced dissolution profiles. It was also observed that the amount of liquid phase in relation to carrier material had an effect on hardness, friability, disintegration of tablet, and tablet height. The use of spraying technique enhanced flow properties of the prepared mixtures, increased hardness, decreased friability, and improved the homogeneity of the final dosage form. Therefore, spraying of a drug in liquid phase onto a carrier in fluid bed equipment seems to be a better preparation method for liquisolid systems as a perspective candidate for clinical usage as dosage forms with improved bioavailability.

## Figures and Tables

**Table 1:** Composition of liquisolid tablets.

Sample	Rosuvastatin		PEG 400		Neusilin US2		Aerosil 200		Kollidon CL-F		Mg stearate		Lactose		*L*_f_^*^
	[mg]	[%]	[mg]	[%]	[mg]	[%]	[mg]	[%]	[mg]	[%]	[mg]	[%]	[mg]	[%]	
40%w	10	1.5	123	18.9	332.5	51.2	6.5	1.0	32.5	5.0	6.5	1.0	139.0	21.4	0.4
50%w	10	1.5	123	18.9	266.0	40.9	5.3	0.8	32.5	5.0	6.5	1.0	206.7	31.8	0.5
60%w	10	1.5	123	18.9	221.7	34.1	4.4	0.7	32.5	5.0	6.5	1.0	251.9	38.8	0.6
70%w	10	1.5	123	18.9	190.0	29.2	3.8	0.6	32.5	5.0	6.5	1.0	284.2	43.7	0.7
80%w	10	1.5	123	18.9	166.4	25.6	3.3	0.5	32.5	5.0	6.5	1.0	308.3	47.4	0.8
90%w	10	1.5	123	18.9	147.8	22.7	3.0	0.5	32.5	5.0	6.5	1.0	327.2	50.3	0.9
100%w	10	1.5	123	18.9	133.0	20.5	2.7	0.4	32.5	5.0	6.5	1.0	342.3	52.7	1.0
110%w	10	1.5	123	18.9	121.0	18.6	2.4	0.4	32.5	5.0	6.5	1.0	354.6	54.6	1.1
120%w	10	1.5	123	18.9	110.8	17.1	2.2	0.3	32.5	5.0	6.5	1.0	365.0	56.2	1.2

^*^ *L*_*f*_ means liquid load factor.

**Table 2:** Flow properties of the tableting mixtures.

Sample	Simple blending				HR	Spraying				HR
	Flowability	Angle of repose	Angle of slide	CI		Flowability	Angle of repose	Angle of slide	CI	
	[s/100 g]	[°]	[°]	[%]		[s/100 g]	[°]	[°]	[%]	
40%w	102.19 ± 0.36	28.06 ± 1.09	36.00 ± 2.00	16.67	1.20	8.88 ± 0.12	29.38 ± 2.36	28.67 ± 1.15	13.08	1.15
50%w	83.16 ± 0.64	27.29 ± 0.46	38.00 ± 2.00	17.53	1.21	7.54 ± 0.61	28.33 ± 1.22	35.67 ± 0.58	14.89	1.18
60%w	63.63 ± 0.49	30.32 ± 0.35	37.67 ± 2.08	17.58	1.21	6.25 ± 0.23	31.21 ± 1.16	29.33 ± 1.53	13.58	1.16
70%w	65.43 ± 0.41	28.97 ± 1.43	36.67 ± 0.58	17.33	1.21	56.37 ± 0.62	26.70 ± 1.28	35.33 ± 0.58	15.38	1.18
80%w	64.80 ± 4.32	26.43 ± 0.48	36.33 ± 0.58	17.02	1.21	57.90 ± 0.90	28.07 ± 0.46	34.33 ± 0.58	15.28	1.18
90%w	60.90 ± 2.70	27.25 ± 0.47	35.67 ± 0.58	16.85	1.20	5.19 ± 0.01	28.57 ± 0.85	38.33 ± 1.53	16.22	1.19
100%w	4.85 ± 0.42	29.40 ± 0.79	35.67 ± 1.15	16.22	1.19	27.72 ± 0.48	30.70 ± 0.88	34.33 ± 1.53	14.29	1.17
110%w	4.60 ± 0.12	31.51 ± 0.87	33.00 ± 1.00	16.90	1.20	32.81 ± 0.98	21.19 ± 0.53	32.33 ± 0.58	15.15	1.18
120%w	4.14 ± 0.34	27.77 ± 0.52	34.33 ± 2.08	18.75	1.23	30.49 ± 1.59	23.83 ± 2.68	33.00 ± 1.00	13.11	1.15

**Table 3:** Pycnometric densities (g/cm^3^) of tableting blends.

Sample	Simple blending		Spraying	
	Density	SD	Density	SD
40%w	1.7765	0.0018	1.7377	0.0039
50%w	1.7639	0.0051	1.6271	0.0020
60%w	1.6088	0.0011	1.6093	0.0023
70%w	1.5854	0.0028	1.5781	0.0024
80%w	1.5530	0.0010	1.5177	0.0013
90%w	1.5318	0.0006	1.5201	0.0028
100%w	1.5441	0.0032	1.5368	0.0025
110%w	1.5269	0.0030	1.5120	0.0055
120%w	1.5340	0.0019	1.5138	0.0006

**Table 4:** Properties of liquisolid tablets prepared by simple blending and spraying.

Sample	Simple blending					Spraying				
	Friability	Disintegration	Uniformity of mass	Drug content	Height	Friability	Disintegration	Uniformity of mass	Drug content	Height
	[%]	[s]	[mg]	[%]	[mm]	[%]	[s]	[mg]	[%]	[mm]
40%w	0.52	3.0 ± 0.0	650.23 ± 5.28	103.01 ± 5.08	8.20 ± 0.07	0.95	18.0 ± 0.0	650.12 ± 3.37	95.58 ± 5.00	6.06 ± 0.05
50%w	0.10	38.0 ± 3.0	649.76 ± 4.25	98.82 ± 7.95	6.48 ± 0.15	0.18	24.0 ± 5.0	657.07 ± 6.70	96.55 ± 1.70	6.22 ± 0.13
60%w	0.06	50.0 ± 3.0	654.43 ± 5.98	99.86 ± 6.66	5.59 ± 0.09	0.04	60.0 ± 13.0	650.50 ± 1.97	106.07 ± 0.77	4.96 ± 0.11
70%w	0.23	159.0 ± 3.0	651.31 ± 4.13	92.29 ± 3.11	4.90 ± 0.04	0.04	98.0 ± 17.0	656.66 ± 6.33	97.35 ± 0.80	4.66 ± 0.06
80%w	0.43	107.0 ± 5.0	655.13 ± 7.71	109.42 ± 4.67	4.73 ± 0.08	0.05	35.0 ± 0.0	656.82 ± 4.30	103.43 ± 4.29	5.05 ± 0.02
90%w	0.56	51.0 ± 0.0	664.31 ± 5.46	66.10 ± 3.83	4.88 ± 0.03	0.03	73.0 ± 0.0	654.74 ± 5.60	95.92 ± 1.89	4.81 ± 0.02
100%w	0.79	39.0 ± 0.0	649.44 ± 3.68	116.76 ± 5.03	4.87 ± 0.03	0.08	30.0 ± 0.0	655.75 ± 0.82	90.55 ± 1.95	4.47 ± 0.17
110%w	0.85	40.0 ± 4.0	659.11 ± 3.29	118.19 ± 13.15	4.94 ± 0.05	0.25	25.0 ± 0.0	652.93 ± 1.70	109.20 ± 1.14	4.61 ± 0.05
120%w	1.45	21.0 ± 0.0	653.12 ± 1.78	85.68 ± 23.70	4.82 ± 0.05	0.16	18.0 ± 0.0	658.05 ± 1.05	112.38 ± 0.92	4.30 ± 0.00

**Table 5:** Water absorption ratio and wetting time.

Sample	Simple blending		Spraying	
	Water absorption ratio	Wetting time	Water absorption ratio	Wetting time
	[%]	[min]	[%]	[min]
40%w	173.17 ± 12.60	3.18 ± 0.03	170.60 ± 15.06	6.32 ± 2.53
50%w	119.93 ± 12.59	4.48 ± 3.02	118.48 ± 6.32	3.38 ± 1.75
60%w	104.22 ± 10.14	7.00 ± 1.12	74.60 ± 3.35	8.70 ± 2.42
70%w	75.26 ± 11.03	12.82 ± 4.92	64.36 ± 9.57	7.25 ± 2.77
80%w	78.10 ± 13.07	26.30 ± 24.52	69.73 ± 15.28	9.37 ± 4.63
90%w	79.98 ± 5.45	25.01 ± 2.28	73.64 ± 8.59	8.07 ± 2.82
100%w	84.19 ± 11.42	31.01 ± 3.37	75.66 ± 5.63	9.93 ± 2.78
110%w	93.58 ± 7.56	52.65 ± 13.27	76.08 ± 7.56	1.67 ± 1.05
120%w	92.94 ± 10.76	55.87 ± 4.77	78.06 ± 3.22	2.87 ± 1.92

**Table 6:** Standard deviation of dissolution studies (%).

Minutes	Simple blending									Spraying								
	40%w	50%w	60%w	70%w	80%w	90%w	100%w	110%w	120%w	40%w	50%w	60%w	70%w	80%w	90%w	100%w	110%w	120%w
5	0.66	3.42	0.71	15.80	3.72	24.27	3.81	10.26	12.79	2.92	3.09	3.37	4.47	1.39	0.64	2.30	3.71	5.15
10	1.60	3.63	2.07	17.00	0.94	23.79	2.32	7.91	13.70	3.32	1.69	2.77	2.06	0.06	1.36	1.46	2.28	2.31
15	1.84	4.20	1.27	17.77	0.90	27.53	3.18	8.33	14.50	3.85	3.72	4.04	1.37	0.25	2.28	1.60	1.44	1.65
20	1.52	4.15	1.66	21.01	1.45	27.39	3.75	8.19	14.97	4.19	0.63	5.90	0.62	0.08	1.64	2.02	0.99	1.03
25	1.58	3.98	1.44	17.89	0.57	25.66	3.83	8.16	15.75	4.61	1.28	6.04	0.55	0.35	0.73	2.26	0.84	0.88
30	1.92	4.68	1.34	17.64	0.60	26.59	3.97	7.48	16.14	5.00	3.48	6.09	0.54	0.24	0.95	2.38	1.14	0.92
